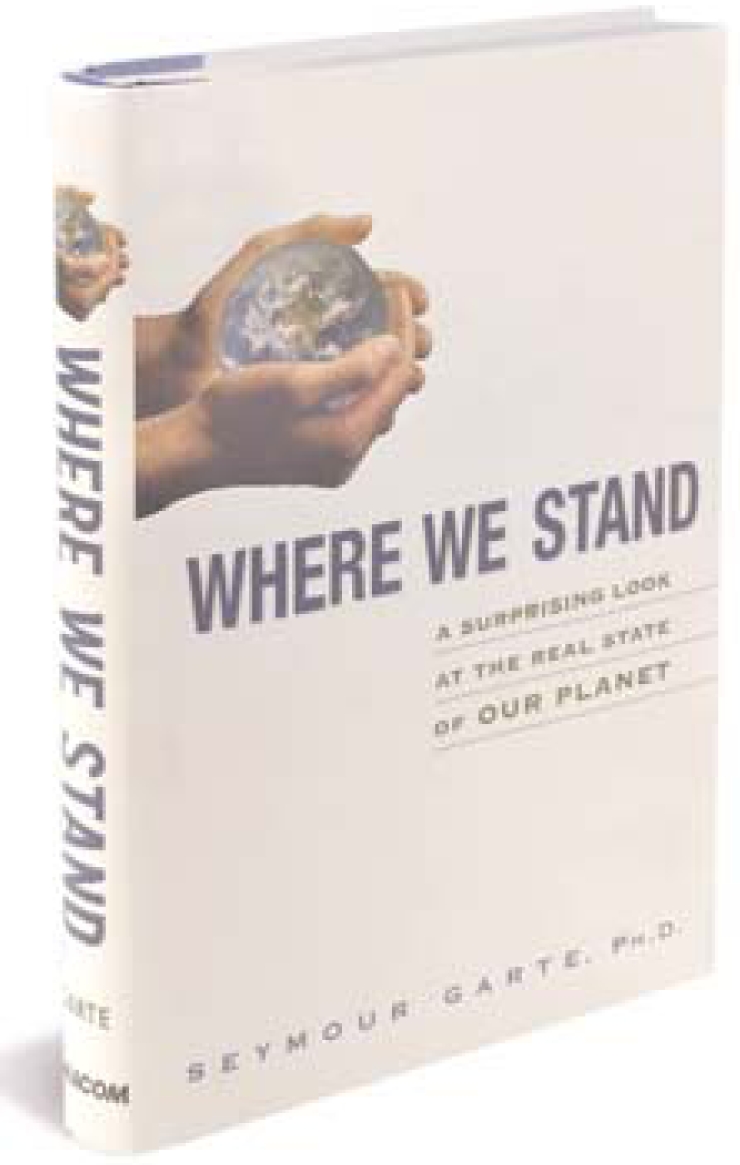# Where We Stand: A Surprising Look at the Real State of Our Planet

**Published:** 2008-06

**Authors:** Paul J. Lioy

**Affiliations:** Paul J. Lioy is a professor and vice chair of the Department of Environmental and Occupational Medicine at UMDNJ-Robert Wood Johnson Medical School, Piscataway, New Jersey. He is deputy director for government relations at the Environmental and Occupational Health Sciences Institute, a joint program of Rutgers University and UMDNJ, and director of the institute’s program in exposure science. He is also an associate editor for EHP.

In an era of pessimism and revisionism, I was able to pick up a book that actually tries to provide a balanced perspective on the state of the environment and the environmental health of our country and other parts of the world. Within 272 pages, Garte followed the progress we have made in cleaning up the environment in the United States and abroad since the enactment of the U.S. Clean Air Act by President Richard Nixon in 1970. What is refreshing about the book’s content is that Garte provides some compelling arguments about these improvements based on the “facts.” What is fascinating about his exposition is that the most of the facts provided, including simple and clear graphics, are readily available for anyone to review. However, I think that few scientists and the general public have truly taken the time to see how much things have improved before professing the next apocalypse.

Garte’s goal was not to paint a rosy picture, but instead to provide a realistic prospective on what has been done since the publication in 1962 of Rachel Carson’s *Silent Spring*, with some cautions about the future. The chapters of *Where We Stand* have distinct sections on progress and, as Garte terms it, “The Bad News.” Having grown up in Passaic, New Jersey, just across the river from Garte’s childhood home, and having worked and lived in the New York/New Jersey metropolitan area for many years, I can attest to the vast improvements in the environment that have occurred over the past 40 years. I am pleased that the book’s content reflects the major successes that have been achieved by the work of many people, including scientists, engineers, regulators, and environmentalists, over that period.

Among the “Historical Lessons” he discusses, Garte provides four case histories—lead, stratospheric ozone, tobacco, and genetically modified organisms—and provides information on how involved parties such as regulatory agencies, research scientists, public health advocates, industries, and the media can interact to deal with these problems. Sometimes progress was not pretty or easy, and Garte achieves a balanced analysis with the way he presents each topic. The section on lead is an excellent example, although Garte did not clearly state that the initial reason for the catalytic converter was the mandate to control the hydrocarbon precursors to tropospheric (sometimes called ground-level) ozone, not just air toxics, especially in California—which in turn led to the phaseout of leaded gasoline. It is also a pity that he did not discuss the unfinished business associated with tropospheric ozone.

Garte also provides an interesting contrast of overarching environmental issues that are not always clearly articulated, and he effectively uses them to explain the difference between where we have been and where we are today in the United States. One of the contrasts he developed was between the growth of Western environmental awareness and action after World War II, and the lack of environmental action during the same period in Eastern Europe under the “Communist Experiment.” He then illustrates some of the progress made in Eastern Europe since the fall of the Berlin Wall in 1989. This entire section alone is worth the purchase price of the book. Further, the contrasts documented from 1960 through 2000 and beyond between the United States and Western Europe, and Eastern Europe and the former Soviet republics would be a useful lecture in any introductory environmental science or environmental health course.

Garte ends the book with details on some, but not all, present and potential future environmental health issues. They clearly will spark debate among readers, and will make some readers question why some issues were included and some not. The conclusion provides an optimistic note and a reaffirmation that humans can be resilient, both individually and collectively, can overcome adversities, and in the end maybe even “behave well.” I share his basic optimism, but I have learned that new challenges, with their consequential opportunities, are always just around the corner.

## Figures and Tables

**Figure:**